# Mediastinal melioidosis: A case series from far North Queensland

**DOI:** 10.1002/rcr2.1017

**Published:** 2022-08-11

**Authors:** Bree‐Anna Gadsby, Andrew Oaten, Phoebe Davies, Graham Simpson, Stephen Vincent

**Affiliations:** ^1^ Department of Thoracic and Sleep Medicine Cairns Hospital Cairns Australia; ^2^ Department of Thoracic and Sleep Medicine Greenslopes Private Hospital Brisbane Australia

**Keywords:** endobronchial ultrasound, mediastinal lymphadenopathy, melioidosis

## Abstract

Melioidosis is the clinical disease caused by the Gram‐negative bacillus *Burkholderia pseudomallei* and is endemic to Northern Australia and Southeast Asia. It is commonly referred to as the ‘great mimicker’ because of its wide range of clinical presentations, often making diagnosis challenging. Isolated mediastinal lymphadenopathy as the presenting feature of melioidosis is rare and can be indistinguishable from tuberculosis or malignancy. Endobronchial ultrasound (EBUS) is the preferred technique for evaluating undifferentiated mediastinal lymphadenopathy but its role in the diagnosis of mediastinal melioidosis remains sparsely reported in the literature. In this case series, we present four cases of mediastinal melioidosis, and the role that EBUS guided fine needle aspiration (FNA) played in the diagnosis and management.

## Graphical abstract

Isolated mediastinal lymphadenopathy as the presenting feature of melioidosis, the clinical disease caused by the Gram‐negative bacillus Burkholderia pseudomallei, is rare and can be indistinguishable from tuberculosis or malignancy. Endobronchial ultrasound (EBUS) is the preferred technique for evaluating undifferentiated mediastinal lymphadenopathy but its role in the diagnosis of mediastinal melioidosis remains sparsely reported in the literature. In this case series, we present four cases of mediastinal melioidosis, and the role that EBUS guided fine needle aspiration (FNA) played in the diagnosis and management.

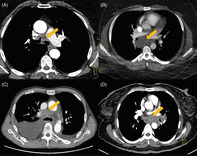

## INTRODUCTION

Melioidosis, an infection caused by the gram‐negative organism *Burkholderia pseudomallei*, is endemic to tropical Northern Australia and Southeast Asia. Whilst it most commonly presents as an acute pneumonic illness with septicaemia, it can involve almost any organ and chronic presentations have been well described.^1^ Mediastinal disease in absence of pneumonia is rare and has only been examined specifically in small case series to date.^2^, ^3^ Within Australia, the Darwin prospective melioidosis series reported 17 cases (of a total of 540 cases) of mediastinal involvement; 12 were associated with pneumonia and 5 were isolated mediastinal disease.^4^ In this case series, we present four cases of mediastinal melioidosis, and discuss the role that endobronchial ultrasound (EBUS) played in diagnostic determination and initiation of appropriate therapy.

## CASE SERIES

### Case 1

A 66‐year‐old Caucasian female was referred to the outpatient Respiratory clinic for further evaluation of a suspected primary lung malignancy. She presented with a 12‐month history of malaise, weight loss and non‐productive cough with a single episode of haemoptysis. Her past medical history was significant for chronic obstructive pulmonary disease, ex‐smoker with a 40‐pack year smoking history, type two diabetes mellitus and dyslipidaemia.

**FIGURE 1:**
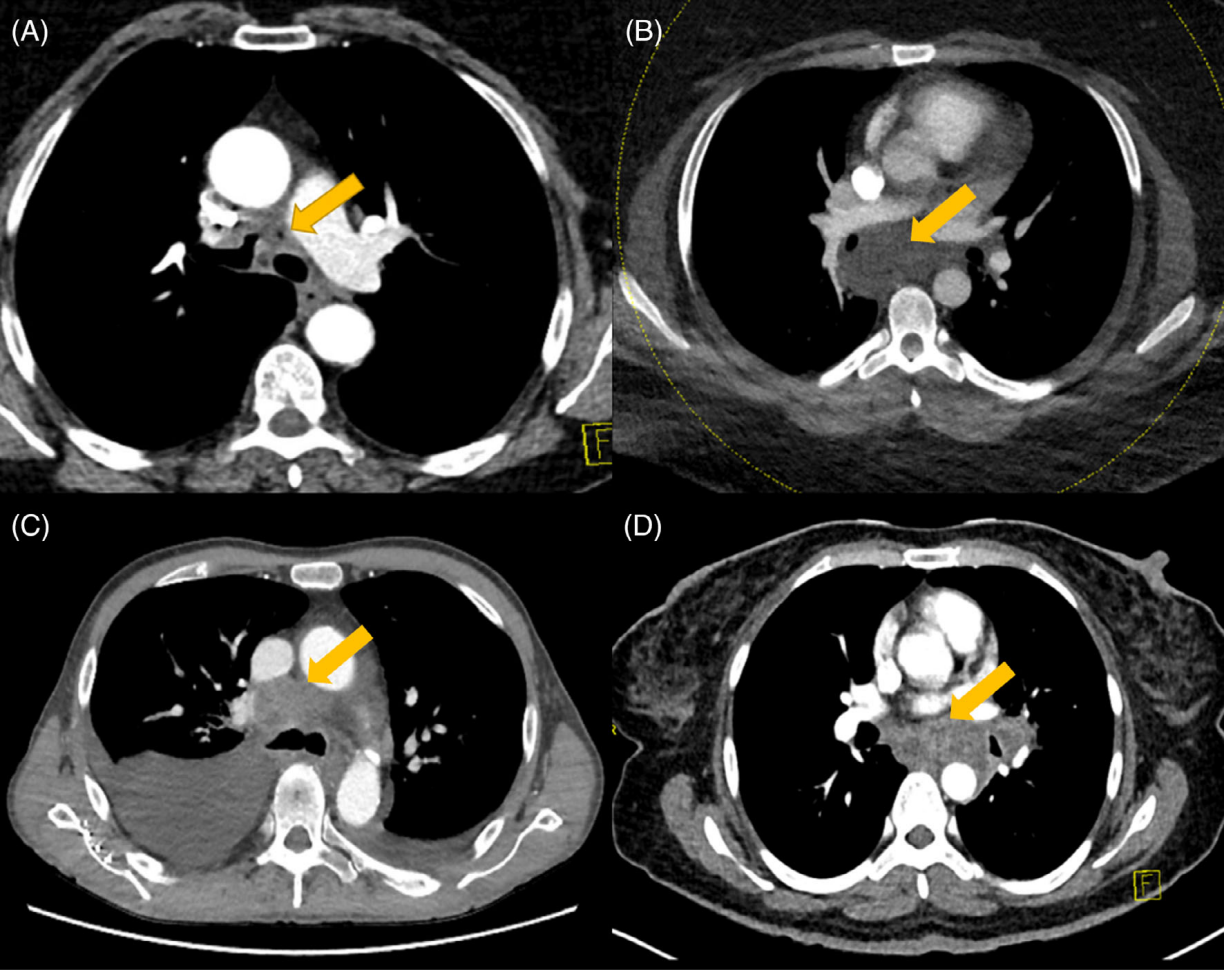
Computed tomography (CT) of the thorax (axial view) for all four patients. (A) Mediastinal lymphadenopathy with fistulous tract between the right main bronchus and sub‐carinal lymph node. (B) Mediastinal lymphadenopathy extending towards the right and encasing the bronchus intermedius. (C) Mediastinal lymphadenopathy and large right sided pleural effusion. (D) Mediastinal and left hilar lymphadenopathy encasing the oesophagus

**FIGURE 2:**
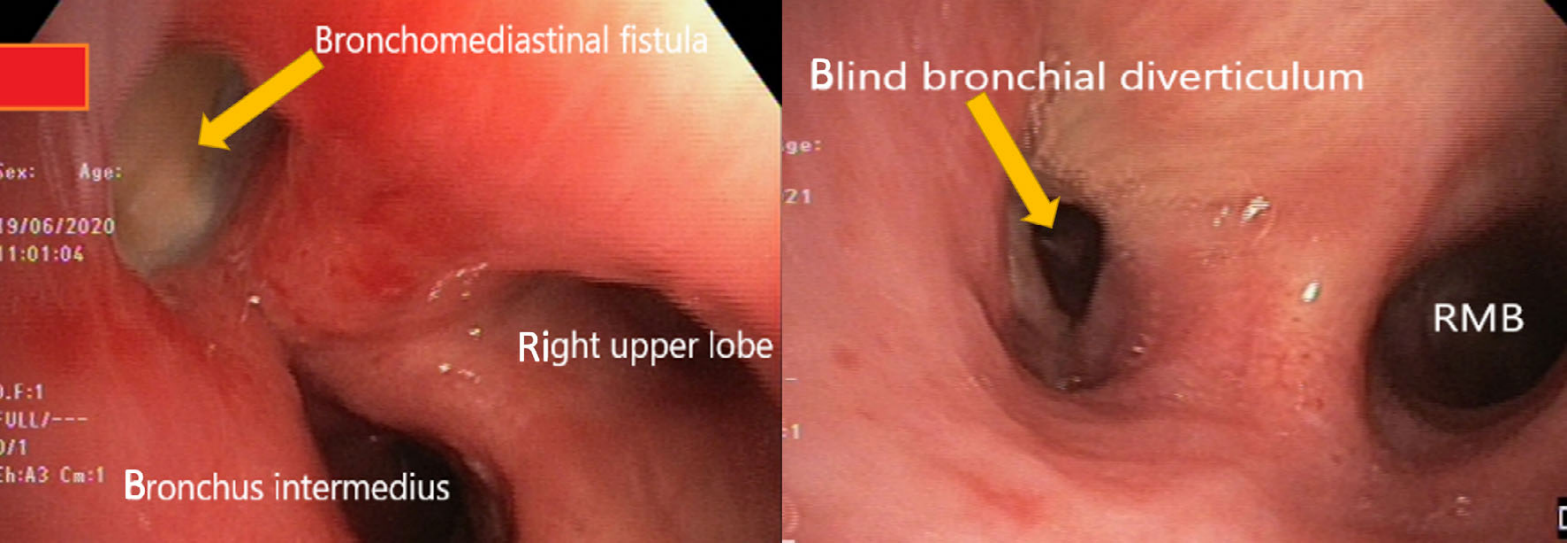
Initial bronchoscopy demonstrating fistulous tract with active infection (left). Repeat bronchoscopy demonstrating partial resolution post antimicrobial therapy (right)

There were no relevant findings on clinical examination or routine blood investigation. Chest imaging with computed tomography (CT) scan demonstrated mediastinal lymphadenopathy with a fistulous tract between the right main bronchus and sub‐carinal lymph node (Figure 1A). Subsequent bronchoscopy revealed necrotic material in the anteromedial wall of the right main bronchus with a fistulous tract (Figure 2). Bronchial washings from the actively infected fistula cultured *B. pseudomallei*.

A positron emission tomography (PET) scan subsequently demonstrated innumerable bilateral pulmonary nodules, with multi‐lobar FDG‐avid pulmonary infiltrates and extensive hilar and mediastinal lymphadenopathy (SUV max of 7.5).

Prolonged antibiotic therapy was required due to mediastinal involvement and persistent growth of *B. pseudomallei* on repeat bronchoscopy 1 month following commencement of treatment. Adequate cure of melioidosis was achieved following a total of 4 months of intravenous and oral antibiotic therapy as per local guidelines. This was determined by resolution of symptoms and improvement in inflammatory markers as well as radiological and bronchoscopic findings.

### Case 2

A 25‐year‐old indigenous female presented to the Emergency Department with a 10‐day history of fevers, headaches, sore throat, productive cough and small volume haemoptysis. She had presented with similar symptoms a few days prior and was treated empirically with oral antibiotics for a mild community acquired pneumonia. Her relevant past medical history included morbid obesity, poorly controlled type two diabetes mellitus, and a recent mild COVID infection which had fully resolved.

On clinical examination, she was tachypnoeic (30 breaths/min), tachycardic (123 beats/min), and febrile (39.2°), but had no localizing findings. Total white cell count (WCC) was 7.0 × 10^9^/L and C reactive protein (CRP) was 184 mg/L. Sputum culture grew normal respiratory commensals and chest X ray was unremarkable. She was treated with intravenous Ceftriaxone and oral Doxycycline for an undifferentiated febrile illness. In spite of intravenous antibiotic therapy, she remained clinically unwell. To further investigate, she had a computed tomography pulmonary angiogram (CTPA) which revealed an abnormal mass behind the pulmonary arteries extending towards the right and encasing the bronchus intermedius with associated right hilar lymphadenopathy that was reported as suspicious for non‐small cell lung cancer (Figure 1B).

EBUS‐guided FNA of the mediastinal mass revealed necrotic material, which was negative on cytology, negative on acid fast bacilli (AFB) stain, but positive for *B. pseudomallei* on bacterial culture. The blood cultures that were collected prior to the procedure showed also isolated *B. pseudomallei*.

The bronchoscopy was complicated by excessive coughing and oxygen desaturation. This required anaesthetic support due to heavy sedation and temporary bag‐valve mask ventilation.

Treatment for melioidosis was commenced according to local guidelines. A repeat CT scan at 1 month showed complete resolution of the mediastinal mass.

### Case 3

A 65‐year‐old Caucasian male presented to the Emergency Department with a 3‐day‐history of fevers, shortness of breath, and productive cough. This is in the setting of a 6‐month history of unintentional weight loss and multiple hospital presentations. His past medical history included for end‐stage renal failure on haemodialysis, coronary artery disease, severe aortic stenosis, multiple melanomatous and non‐melanomatous skin cancers requiring excision. He was an ex‐smoker with an 8‐pack year history.

On clinical examination, he was tachypnoeic (20 breaths/min) and borderline hypoxic (SpO_2_ 93% on room air). Except for an ejection systolic murmur and bibasal crackles, systemic examination was otherwise normal.

Sputum culture grew scant gram‐negative bacilli and blood culture had no growth. CT imaging of the chest demonstrated mediastinal lymphadenopathy and bilateral pleural effusions. This was reported as being suspicious for malignancy – either metastatic melanoma or squamous cell carcinoma (Figure 1C). A thoracocentesis demonstrated a transudative pleural effusion (pleural fluid protein 13 g/L and LDH 81 unit/L) with negative cytology and bacterial cultures. EBUS guided FNA of the mediastinal lymph nodes cultured *B. pseudomallei* and the patient was treated for melioidosis as per local guidelines. Notably, the bronchoscopy was complicated by massive haemoptysis, and required emergent repeat bronchoscopy with argon plasma coagulation (APC) and blood transfusion.

Repeat CT imaging at 1 month then 4 months demonstrated ongoing but improving mediastinal lymphadenopathy. Due to concerns about persistent melioidosis, the eradication phase of therapy was extended from 3 to 6 months. During this time, he suffered from a cardiac arrest and passed away, but this was thought to be related to his underlying coronary artery disease.

### Case 4

A 36‐year‐old indigenous female presented to the Emergency Department with a 2‐week history of fevers and non‐productive cough. This was in the context of a 6‐month history of malaise, anorexia and unintentional weight loss. She had presented on two separate occasions in the preceding month for similar symptoms and had been discharged on empiric oral antibiotics. Her past medical history was significant for poorly controlled type two diabetes mellitus and active smoker with a 23‐pack year history.

On clinical examination, she was tachypnoeic (28 breaths/min) and febrile (38.1°). Her systemic examination and routine bloods were normal except for a CRP of 114 mg/L. Chest radiograph revealed a bulky left hilum and subsequent CT chest demonstrated left hilar lymphadenopathy and subcarinal lymphadenopathy, encasing the oesophagus (Figure 1D). CT imaging was reported as possible primary left bronchial neoplasm – suspicious for small cell lung cancer.

The pre‐test probability of a primary bronchogenic malignancy was high, and she was listed for a bronchoscopy with EBUS. Prior to the procedure, sputum culture returned a positive result for *B. pseudomallei* and the procedure was cancelled. Treatment for melioidosis was commenced according to local guidelines. Follow‐up CT imaging at 1 month demonstrated complete resolution of lymphadenopathy.

## DISCUSSION

There were several important similarities identified between our patients who had a diagnosis of mediastinal melioidosis. Three out of four patients had chronic presentations (defined as symptoms present for greater than 2 months) and all four patients had recognized risk factors, including diabetes mellitus, chronic lung disease and chronic kidney disease.^1^ In all four cases, there was no clear identified occupational or recreational inoculation event. Characteristically, two out of four patients presented during the wet season.^4^ In all cases where EBUS‐guided FNA was performed, there was a positive culture confirming *B. pseudomallei*. Two out of four patients had a prompt response to therapy with complete resolution of findings on repeat imaging following the intensive phase of therapy. Our local treatment guidelines recommend an intensive intravenous phase for at least 2 weeks followed by an eradication oral phase for at least 3 months. Only one patient had a disease‐related complication which was formation of a broncho‐mediastinal fistula that partially resolved with antimicrobial therapy. However, this defect is unlikely to be of significance. One patient died during treatment, but this was related to his underlying coronary artery disease.

Isolated mediastinal lymphadenopathy as the presenting feature of melioidosis is rarely described in the literature. These unusual presentations often lead to delays in diagnosis and treatment, as seen in our cases. The gold standard for diagnosis is isolation of the bacteria by culture.^1^ However, in the setting of mediastinal lymphadenopathy without pulmonary involvement, cultures from blood, sputum, or bronchoalveolar lavage may be insufficient. This was true for two out of four of our patients. EBUS‐guided FNA is the preferred technique for evaluating and obtaining tissue samples from mediastinal lesions,^5^ but its role remains under‐investigated and sparsely reported in the diagnosis of mediastinal melioidosis. We found that EBUS‐guided FNA aided in the diagnosis in all cases where it was performed. This was also appreciated in two case series in India where blood and sputum cultures remained negative.^2^, ^3^ While bronchoscopy is generally a safe procedure, two out of three patients in our case series experienced procedure‐related complications. These included significant haemoptysis requiring emergent repeat bronchoscopy and blood transfusion as well as excessive coughing requiring heavy sedation and anaesthetic support. In addition, bronchoscopy was avoided in one patient where sputum culture provided a diagnosis. The significance of the positive sputum culture in the absence of pneumonia is uncertain but may indicate endobronchial disease not detectable on imaging. We feel that this leaves open a clinical question regarding the timing of bronchoscopy in the diagnostic work up.

Mediastinal melioidosis can mimic the presentation of primary lung malignancy.^6^, ^7^, ^8^ In each of the four cases presented, a presumptive diagnosis of malignancy had been made and this suspicion was communicated to patients. Factors which contributed to this diagnostic bias include the smoking history, the chronicity of symptoms, and the radiological reporting of chest imaging findings. There are obvious psychosocial impacts when disclosing a suspected lung malignancy diagnosis to patients and their families. In addition, the ongoing diagnostic workup can be slow to progress, resulting in further anxiety and distress to patients.

In conclusion, whilst isolated mediastinal melioidosis appears to be a rare presentation, it bears consideration in diagnostic workup for mediastinal lymphadenopathy in parts of the world where melioidosis is endemic. These cases underscore the important role that EBUS‐guided FNA with adequate culture samples can play in workup of mediastinal masses, particularly for infections, that are otherwise difficult to isolate.

## AUTHOR CONTRIBUTION

Bree‐Anna Gadsby, Andrew Oaten and Phoebe Davies contributed to conception of the work and drafting the article. All authors contributed to critical revision of the article and have provided final approval of the version to be published.

## CONFLICT OF INTEREST

None declared.

## ETHICS STATEMENT

The authors declare that appropriate written informed consent was obtained for the publication of this case series and accompanying images.

## Data Availability

The data that support the findings of this study are available from the corresponding author upon reasonable request.
